# AWaRe classification of antibiotics prescribed within 2018-2021 for hospitalised medical and surgical patients in Uyo, Nigeria

**DOI:** 10.11604/pamj.2023.46.80.33027

**Published:** 2023-11-09

**Authors:** Agantem Ekuma, Asukwo Onukak, Sylvanus Udoette, Ann Versporten, Ines Pauwels, Oyinlola Oduyebo, Herman Goossens

**Affiliations:** 1 Department of Medical Microbiology and Parasitology, University of Uyo, Uyo, Nigeria,; 2 Department of Internal Medicine, University of Uyo Teaching Hospital, Uyo, Nigeria,; 3 Laboratory of Medical Microbiology, Vaccine and Infectious Disease Institute, Faculty of Medicine and Health Sciences, University of Antwerp, Antwerp, Belgium,; 4 Department of Medical Microbiology and Parasitology, College of Medicine, University of Lagos, Lagos, Nigeria

**Keywords:** Point prevalence surveys, antimicrobial stewardship, antimicrobial resistance, essential medicines list, AWaRe classification, antibiotics, prescription, tertiary hospital, patients

## Abstract

**Introduction:**

point prevalence surveys have been used as a standardized tool to monitor antibiotic consumption to inform antimicrobial stewardship interventions in many countries. The 2021 WHO model list of Essential Medicines has classified antibiotics into three groups: access, watch and reserve. The aim of this paper is to describe the antibiotics used within a space of three years between 2018 and 2021 at the University of Uyo Teaching Hospital based on WHO AWaRe classification.

**Methods:**

three point-prevalence surveys were conducted in the wards in our 500-bed tertiary hospital in 2018, 2019 and 2021. Each ward was surveyed on a particular day within a four-week period. The wards were grouped into medical and surgical for comparison. Antibiotics were classified as access, watch, and reserve. Validated data were analyzed with IBM SPSS Statistics for Windows, Version 20.0 (Armonk, NY: IBM Corp.).

**Results:**

a total of 526 patients were surveyed out of which 344 were on antimicrobial therapy with a total of 687 antibiotic prescriptions. The overall prevalence of patients who received at least one antimicrobial was 65.4% (62.4 -72.8%). The Access group of antibiotics made up 48.2% of prescriptions while the watch group made up 50.5% of prescriptions. More watch Antibiotics were prescribed by surgical wards (49.7%) than by medical wards (43.7%).

**Conclusion:**

the use of Access group antibiotics in our hospital falls below the WHO target level in both medical and surgical wards. There is a need for strengthening antibiotic stewardship activities to reduce the use of watch group antibiotics and limit antimicrobial resistance.

## Introduction

Antimicrobial resistance has emerged as an important threat to public health globally and in Nigeria [1]. Misuse and overuse of antibiotics create selective pressure, allowing resistant strains to proliferate and spread. There is a worldwide increase in antibiotic consumption with most of the increase occurring in low and middle-income countries [2]. Although there is paucity of data on the scope of AMR in Nigeria, a recent government report has noted an untenably high rate of drug-resistant pathogens [3].

The World Health Organization (WHO) has identified antimicrobial resistance as a major threat due to the lack of new antibiotics in the development pipelines and infections caused by multidrug resistant pathogens becoming untreatable [4]. Point prevalence surveys (PPS) have been used as a standardized tool to monitor antibiotic consumption to inform antimicrobial stewardship interventions in many countries [5]. The 2021 WHO model list of Essential Medicines has classified 180 antibiotics available worldwide into three groups; access, watch and reserve [6]. The Access group contains antibiotics active against a wide range of pathogens while showing lower resistance potential than antibiotics in other groups. They should be widely available, affordable and quality assured. watch group include antibiotics with a higher potential for resistance. They are recommended as essential first or second choice empiric treatment options for a limited number of specific infectious syndromes and should be prioritized as key targets of antimicrobial stewardship programmes and monitoring. The reserve group includes “last resort” antibiotics that should be reserved for treatment of confirmed or suspected infections due to multi-drug-resistant organisms [7].

**Objective:** the aim of this paper is to describe the AWaRe antibiotics use pattern based on results from three-point prevalence surveys carried out between 2018 and 2021 in a tertiary hospital in Uyo, Nigeria to determine the trend of antibiotic prescribing and for antimicrobial stewardship.

## Methods

**Study design and setting:** three point-prevalence surveys were conducted in our 500-bed tertiary hospital in 2018, 2019 and 2021. This hospital provides specialist care in Internal Medicine, Surgery, Paediatrics and Obstetrics/Gynaecology and other subspecialties to Akwa Ibom State neighbouring state with an average of over admissions each month. Each ward was surveyed on a particular day within a four-week period.

**Participants/study size:** all the patients on admission at 08: 00h on the day of the survey were included.

**Variables:** all the wards were surveyed in 2018 while randomly selected adult medical and surgical wards as well as pediatrics and obstetrics and gynecology wards were surveyed in 2019 and 2021. The wards were grouped into medical and surgical wards for comparison. Outpatients were excluded.

**Data sources:** the questionnaire gathered information on basic patient demographics, antimicrobial agents used, indication for treatment, laboratory data prior to treatment, stop/review date, whether reason was stated in notes and compliance of prescription to guideline.

**Quantitative variables:** Antibiotics were classified as access, watch, and reserve on the basis of the 2021 WHO AWaRe classification [7]. Fixed-dose combinations of broad-spectrum antibiotics were grouped in a fourth category: 'Not recommended'. Only antibiotics for systemic use (ATC J01) and oral nitroimidazole derivatives (P01AB) were included in the prescription-level analyses. Antimycotics (J02), antimalarials (P01B), antivirals (J05) and intestinal anti-infectives (A07) were excluded.

**Statistical methods:** patients' data were collected on paper forms and entered into the Global PPS web-based tool for data entry and validation designed by the University of Antwerp (Antwerp, Belgium). The Global Point Prevalence Survey uses standardized, pretested and validated tools [8]. Validated data were exported to Microsoft Excel (Microsoft Corp., Redmond, WA) and were analyzed with IBM SPSS Statistics for Windows, Version 20.0 (Armonk, NY: IBM Corp.).

**Ethical approval:** this was obtained from the Research and Ethics Committee of the University of Uyo Teaching Hospital (NHREC/24/06/22/UUTH).

**Funders for the study:** the Global Point Prevalence Survey is coordinated at the University of Antwerp, Belgium and sponsored through an unrestricted grant given to them annually by bioMérieux. However, the hospital did not receive any funding support for participating in the survey. The funder had no role in the study design, as well as in the data collection, analysis or interpretation, or in the drafting of the manuscript.

## Results

**Participants:** a total of 526 patients were surveyed during the three years under study: (197 in 2018, 226 in 2019 and 103 in 2021) out of which 344 were on antimicrobial therapy with total of 687 antibiotics prescriptions. The overall prevalence of patients that received at least one antimicrobial was 65.4% (62.4 -72.8%).

**Descriptive data:** the mean age, age and gender distribution of the patients are shown in Table 1.

**Table 1:** characteristics of surveys

Characteristics	2018	2019	2021	Total
Total no of patients surveyed	197	226	103	526
Total no of patients on antimicrobial therapy	123	146	75	344
Mean age of patients on antimicrobial therapy (years)	35.9 ± 19.7	36.8 ± 20.3	39.3 ± 14.8	37.1 ± 18.9
Adult	86	107	70	263
Female	64	87	42	193
Antimicrobial prevalence	62.4	64.6	72.8	65.4
Total no of antibiotics prescriptions	242	304	141	687

**Outcome data:** Table 2 shows the AWaRe classification of prescribed antibiotics during the three PPS. The access group of antibiotics made up 48.2% of prescriptions while the watch group made up 50.5% of prescriptions. No reserve group antibiotic was prescribed during the period of the PPS although 1.3% prescription were in the Not Recommended group of antibiotics which were combinations of penicillins like ampicillin/cloxacillin and ceftriaxone/beta-lactamase inhibitor combinations.

**Table 2:** prescribed antibiotics by AWaRe classification

	Year							
	2018		2019		2021		Total	
**AWaRe classification**	Number	Percentage (%)	Number	Percentage (%)	Number	Percentage (%)	Number	Percentage (%)
Access	118	48.8	151	49.7	62	44.0	331	48.2
Watch	124	51.2	144	47.4	79	56.0	347	50.5
Reserve	0	0	0	0	0	0	0	0
Not recommended	0	0	9	2.9	0	0	9	1.3
Total	242	100	304	100	141	100	687	100

**Figure 1:**
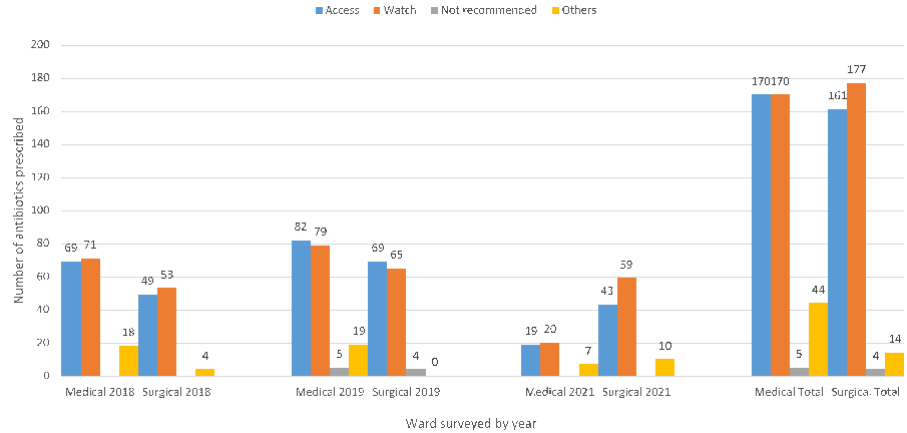
number of antibiotics prescribed in medical and surgical wards using WHO AWaRe classification by year

**Main result:** more watch antibiotics were prescribed by surgical wards (49.7%) than by medical wards (43.7%). The prescription of access antibiotics was similar in both medical (43.7%) and surgical (45.2%) wards as represented in Figure 1.

**Other analyses:** skin and soft tissue infections accounted for 18.2% of watch prescriptions, followed by prophylaxis for Obstetrics/Gynaecological surgery (16.4%) and sepsis (8.6%) as shown in Table 3, which represents the indications for use of watch antibiotics.

**Table 3:** commonest indications for watch antibiotics use

Indication	Percentage of all Watch antibiotics
Skin and soft tissue infections	18.2%
Prophylaxis for Obstetrics/Gynaecological surgery	16.4%
Sepsis	8.6%
Prophylaxis for Bone/Joint procedures/conditions	8.4%
Prophylaxis for Gastrointestinal surgery/conditions	8.4%
Pneumonia	6.9%
Intra-abdominal sepsis	3.2%
Obstetrics/Gynaecological infections	3.2%
Central nervous system infections	2.9%
Ear, nose and throat infections	2.6%

## Discussion

In this study we used the AWaRe classification to describe the pattern of antibiotic consumption in our hospital between 2018 and 2021. The access group of antibiotics has the lowest resistance potential compared to the other groups. Some of the drugs are the first line or second line drugs recommended by the WHO for the treatment of several infections [9]. Compared to the WHO target of at least 60% use of access class antibiotics in every country [10], we found only 48.2% utilization of access group of antibiotics. This pattern of use of access antibiotics was similar in both the medical and surgical specialties. Although higher rates of access antibiotic use have been reported from other African countries [11], the rate in our study falls within the global regional average which ranges from 28.4% in West and Central Asia to 57.7% in Oceania [12]. A global, longitudinal analysis of national pharmaceutical sales data has shown that the increase in consumption of watch antibiotics between 2000 and 2015 was more pronounced in low- and middle income countries (LMICs) and the proportion of countries in which Access antibiotics constituted at least 60% of total consumption decreased substantially between 2000 and 2015 [13].

The low rate of consumption of the access drugs may largely be due to the non-availability of some of these basic drugs and is also a reflection of the challenges in the health systems of developing countries such as Nigeria. There is usually an inverse relationship between the income level of the country and the availability of essential antibiotics [9]. Perceived ineffectiveness of some access group antibiotics may have contributed to the unavailability of these drugs by causing reduced demand. Countries that are far from attaining universal health coverage are likely to have poor access to some essential antibiotics [14]. A study on evaluating antibiotics availability and usage in 20 LMICs found that 17 access and watch antibiotics were stocked by less than a median of 50% of facilities [15]. However, while the WHO target of 60% is about total national antibiotic use, our study focused on a tertiary hospital's antibiotic use where the severity of infections may also have influenced lower usage of access drugs.

We observed a high use of watch antibiotics across the three PPS. The use of watch antibiotics was slightly higher in surgical wards, and this may be due to the use of this group of antibiotics for surgical prophylaxis. The widespread use of watch group of antibiotics, like ceftriaxone, for surgical prophylaxis may be due to lack of guidelines and low confidence in first generation cephalosporins coupled with their unavailability. This trend can increase selection pressure and worsen antimicrobial resistance rates [11]. The watch group of antibiotics consists about 110 drugs with a broader spectrum of activity than access antibiotics and should usually be second line antibiotics for some indications. Inappropriate use of the watch group of antibiotics has a higher probability of developing resistance [15,16].

When compared by the overall volume, the three more common indications for the watch group of antibiotics in our study were skin and soft tissue infections, prophylaxis for obstetrics/gynecological surgery and sepsis. The Infectious Diseases Society of America recommends the use of mostly access antibiotics like cefazolin, clindamycin, dicloxacillin and doxycycline in the treatment of mild to moderate skin and soft tissue infections, whether purulent or non-purulent [17], while watch and reserve group antibiotics are preserved for severe infections. A review by Clifford *et al*. on the prophylactic use of antibiotics in obstetrics/gynecological procedures has also recommended different combinations of access antibiotics like cephazolin, metronidazole and doxycycline as the first line for various specific procedures [18].

There is therefore a need for antibiotic guidelines backed by reliable local antibiogram to guide empiric antibiotic utilization, thereby limiting the use of watch antibiotics in similar settings in LMICs [19]. Two-thirds of the antibiotics prescribed in cases where the indication for antibiotic prescription was uncertain were watch group antibiotics. This underscores the high rates of empirical antibiotic prescriptions due to lack of timely laboratory investigations which has been noted in Nigeria [20,21]. Campaigns targeting persons seeking care in hospital can also help reduce the demand for antibiotic prescription [16]. Giving priority to access antibiotics on the national essential drug list is another strategy that have been used to reduce consumption of watch antibiotics [22]. There is also an urgent need for a globally coordinated guidance on the use of watch and reserve antibiotics in order to contain the threat posed by antimicrobial resistance worldwide [22].

There was no utilization of reserve antibiotics in our study. This is in keeping with data from other countries [12]. While hoping that this was due to efforts at a more efficient antibiotic use, the high rate of multidrug resistant organisms in Nigeria suggests that this may rather reflect the unavailability and/or high cost of this class of antibiotics [23].

The AWaRe classification of antibiotics is designed to enhance antimicrobial stewardship as it provides an effective tool for monitoring the usage of antibiotics. The classification system places various antibiotics into groups based on their clinical usefulness and the potential for the development of resistance when they are used [24]. The AWaRe classification has enabled standardized data collection on antimicrobial consumption across several regions and countries making comparisons easier and also creating avenues for the development of globally applicable stewardship programmes [9]. While most studies on antimicrobial consumption emanate from high income countries, indiscriminate use of antibiotics is widespread in low- and middle-income countries. This situation in the low income countries has been attributed to relatively easy access to unprescribed antibiotics, poor drug regulations and insufficient enforcement of laws to prevent unauthorized access to antibiotics [16,24]. Other factors that also contribute to the improper use of antibiotics in resource-poor settings include high burden of infectious diseases and insufficient diagnostic tools for infections making clinicians over reliant on empirical antibiotics [22]. This study provides information on the AWaRe classification of antibiotic prescriptions in a resource-limited setting. Trends in antimicrobial use over time can serve as an evaluation tool for the antibiotics stewardship programmes at both regional and national level [24].

There were some limitations in this study. Not all the wards in the hospital were assessed in 2019 and 2021 PPS. Also the PPS was carried out at different months of the year; in 2018 it was conducted in February to March, in 2019, October-November, and in 2021, February. Antibiotic consumption rates may vary between different months of the year due to seasonal incidence of some infections. Furthermore, only the antibiotic consumption of admitted patients was evaluated in this study. The exclusion of outpatients from PPS may underestimate the extent of inappropriate antibiotics usage in Nigeria. Some studies have reported that widespread inappropriate use of antibiotics occurs in communities and outpatient settings in several middle and low income countries [12,16]. Data on antibiotics prescribing rate in children formed less than 25% of this study. The relatively small number of children included in this study may also not be representative of the actual rate and pattern of antibiotics prescribed for children. Future surveys with an emphasis on the pediatric population will be required to address this need.

## Conclusion

The use of access group antibiotics in our hospital falls below the WHO target level in both medical and surgical swards. There is need for strengthening of antibiotic stewardship activities to reduce the use of watch group antibiotics and limit antimicrobial resistance.

### What is known about this topic

- *Point prevalence surveys have been used as a standardized tool to monitor antibiotic consumption*;
- *The 2021 WHO model list of Essential Medicines has classified antibiotics into three groups; access, watch and reserve*;
- *WHO has set a target of at least 60% use of access class antibiotics in every country*.

### What this study adds

- *The access group of antibiotics made up 48.2% of prescriptions in our tertiary hospital study*;
- *The use of watch group of antibiotics was higher in surgical wards due to their use for surgical prophylaxis*.
